# Collagen XVI Induces Expression of *MMP9* via Modulation of AP-1 Transcription Factors and Facilitates Invasion of Oral Squamous Cell Carcinoma

**DOI:** 10.1371/journal.pone.0086777

**Published:** 2014-01-23

**Authors:** Konstanze B. Bedal, Susanne Grässel, Peter J. Oefner, Joerg Reinders, Torsten E. Reichert, Richard Bauer

**Affiliations:** 1 Department of Oral and Maxillofacial Surgery, University Hospital Regensburg, Regensburg, Germany; 2 Department of Orthopaedic Surgery, Experimental Orthopaedics, University Hospital Regensburg, Regensburg, Germany; 3 Center for Medical Biotechnology, BioPark I, Regensburg, Germany; 4 Institute of Functional Genomics, University of Regensburg, Regensburg, Germany; Wayne State University, United States of America

## Abstract

Collagen XVI belongs to the family of fibril-associated collagens with interrupted triple helices (FACIT). It is overexpressed during the progression of oral squamous cell carcinoma (OSCC). The present data show a strong collagen XVI-dependent induction of *MMP9* and an increase in OSCC cell invasion. We found activated integrin-linked kinase (ILK) in a complex with kindlin-1 and activation of protein kinase B (PKB/Akt) to be responsible for *MMP9* induction. Inhibition of the formation of focal adhesions reduced MMP9 expression. Moreover, collagen XVI overexpressing OSCC cell clones (COLXVI cell clones) transfected with vectors containing different *MMP9* promoter fragments adjacent to a luciferase reporter revealed an increase in luciferase signal dependent on AP-1 binding sites. Deletion of the AP-1 binding site 98 bp upstream of the reported transcription start site and inhibition of AP-1 with Tanshinone IIA resulted in decreased MMP9 expression. The AP-1 subunit JunB showed differential expression between COLXVI cell clones and mock control cells. Additionally, mass spectrometric analysis of immunoprecipitates revealed that c-Fos interacted strongly with dyskerin in COLXVI cell clones compared to mock controls.

## Introduction

Oral squamous cell carcinoma (OSCC) is by far the most common form of head and neck cancer [1]. Its incidence has increased sharply over the last 10 years. Despite continued improvements in surgery, chemotherapy and radiation therapy, the 5-year survival rate is still only about 50% [2]. This is due to the fact that malignant oral keratinocytes show a fast invasion of cervical lymph nodes and spread quickly to distant sites [3]. Our understanding of the molecular factors responsible for the strong invasive, migratory and proliferative activity of OSCC cells is still incomplete. Here, we present data that imply a critical role of collagen XVI in OSCC invasion.

Collagen scaffolds in tumors are severely altered due to an imbalanced expression of critical extracellular matrix (ECM) components, thereby promoting cancer as they affect metabolic activity and cell signalling [4]–[6]. Collagen XVI is a FACIT collagen (fibril associated collagen with interrupted triple helices). In normal skin, collagen XVI is incorporated into structurally and functionally discrete matrix aggregates that are localized in the dermal-epidermal junction zone of the papillary dermis [7], [8]. Collagen XVI plays an active role in anchoring microfibrils to basement membranes. It is not only produced by dermal fibroblasts but also by smooth muscle cells [9], dermal dendrocytes [10], articular and costal chondrocytes [7], endometrial stromal cells [11], basal dermal and oral keratinocytes [8], [12], [15], neurons from the dorsal root ganglion [13] and glioblastoma / astrocytoma cells [14]. Recent studies have shown, that collagen XVI is implicated in the development of glioblastoma and OSCC, in which it is overexpressed during tumor progression and influences cell cycle progression [12], [14]–[16].

Here, we show the collagen XVI dose-dependent induction of MMP9 in OSCC via integrin-linked kinase (ILK) and protein kinase B (PKB/Akt). In the presence of excess collagen XVI, both kinases were strongly activated and led to an induction of *MMP9* promoter activity. We found the AP-1 binding site at 98 bp upstream of the start codon of *MMP9* to be responsible for the induction. Closer analysis revealed that collagen XVI modulates c-Fos/JunB expression and protein interaction partners via the integrin/ILK/PKB/Akt signalling axis eventually leading to enhanced MMP9 expression and invasion of OSCC cells.

## Results

### Induction of full-length collagen XVI expression in an OSCC cell line

**Figure 1:**
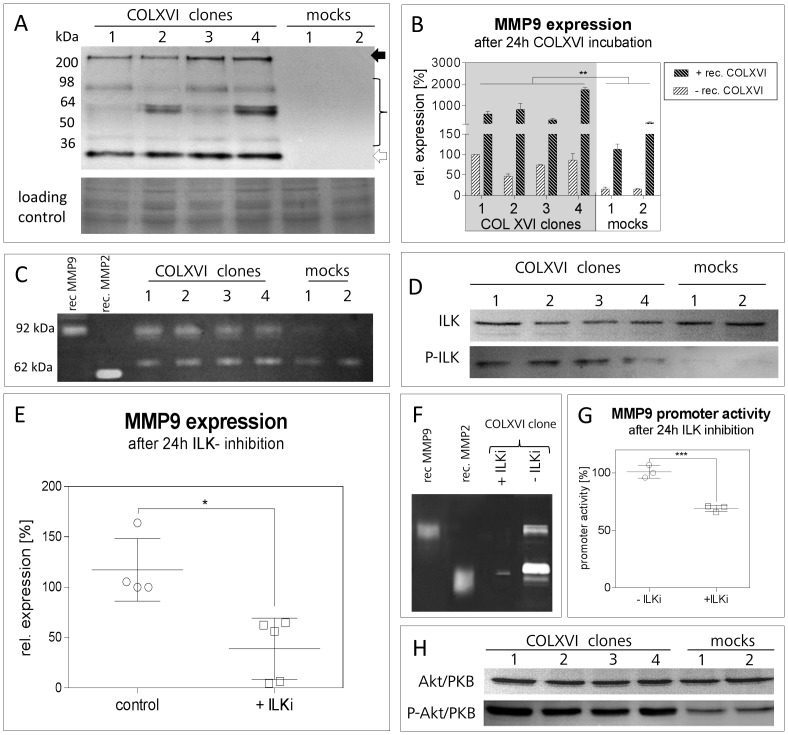
COLXVI overexpression induces MMP9 expression. (**A**) Immunoblot analysis of collagen XVI secretion in supernatants of COLXVI cell clones (clones 1-4) and mock control cells (mock 1-2). Only COLXVI cell clones secret the full-length form of COLXVI (213 kDa; black arrow). Clones 3 and 4 exhibit higher COLXVI secretion than clones 1 and 2. COLXVI cell clones also secrete collagen XVI fragments. A Coomassie Blue membrane staining was used as loading control. (**B**) Quantitative PCR of *MMP9* expression in COLXVI cell clones and mocks after 24 h incubation with/without recombinant collagen XVI. COLXVI cell clones (1-4) show a significant expression of *MMP9* that is further enhanced by the addition of recombinant collagen XVI (n = 3). (**C**) Gelatin zymography of COLXVI cell supernatant and mock controls. The COLXVI cell clones (1-4) show a clear gelatinolytic activity at 92 kDa (pro form). In contrast, the mock control cells (1-2) show very weak MMP9 bands. (**D**) Immunoblot of total ILK and phosphorylated ILK (P ILK) isolated from membrane fractions of COLXVI cell clones and mock control cells. ILK is activated in COLXVI cell clones whereas in mock control cells P-ILK is lacking. (**E**) Quantitative PCR of *MMP9* expression after ILK inhibition with Cpd 22 (ILKi). The expression of *MMP9* decreased after ILK inhibition in the COLXVI cell clones. (n = 3). (**F**) Gelatin zymography of the supernatant of the COLXVI cell clone 3 after ILK inhibition with Cpd 22 (ILKi; c  =  300nM). After ILK inhibition the COLXVI cell clone depicts decreased MMP9 secretion. (**G**) Promoter activity of the COLXVI cell clone 3 after ILK inhibition with Cpd 22 (ILKi; c  =  300 nM). ILK inhibition results in a significant decrease of the MMP9b promoter activity. (p<0.001; n = 3). (**H**) Immunoblot of total Akt/PKB and phosphorylated Akt/PKB (P-Akt/PKB) in cell lysates of COLXVI cell clones (clones 1-4) and mock control cells (1-2). Akt/PKB activation is increased in COLXVI cell clones compared to mock controls.

The OSCC cell line PCI13, which is essentially devoid of endogenous collagen XVI expression, was stably transfected with the coding DNA sequence of full-length collagen XVI. We generated four collagen XVI overexpressing cell clones (COLXVI cell clones) and two mock control clones (empty vector only). The COLXVI cell clones showed different levels of expression and secretion of full-length collagen XVI, while the mock control cells did not express collagen XVI (figure 1A). In COLXVI cell clones we observed additional truncated forms of collagen XVI. We observed an 80 kDa form, which has been described previously by Kassner et al. [17]. In addition, we found processed bands of collagen XVI with molecular weights of 60 kDa and 40 kDa, as well as a band with the size of the NC11 domain of collagen XVI (30–35 kDa, confirmed by mass spectrometry (unpublished data)) (figure 1A). To reveal potential dose-dependent effects of collagen XVI, the collagen XVI low and high expressing clones 1 and 3 were chosen for further experiments.

### Collagen XVI induces MMP-9 expression

Quantitative RT-PCR revealed an eight-fold induction of MMP9 expression in COLXVI cell clones compared to mock controls (figure 1B). Treating COLXVI and control cells with additional (1 µg/mL) recombinant collagen XVI augmented the already strong increase in MMP9 expression (figure 1B). These data were confirmed by gelatin zymography experiments showing strongly increased MMP9 secretion in COLXVI cell clones (figure 1C). We also observed an about 2-fold increase of MMP1 and MMP2 gene expression in COLXVI cell clones (data not shown).

### Collagen XVI overexpression leads to activation of ILK

In a previous publication we have shown that collagen XVI exerts its effects via activation of beta1 integrin and kindlin-1 [12]. As it is known that the activation signal from integrin beta1 is transmitted intracellularly via integrin-linked kinase (ILK) [18], we tested if ILK was activated by phosphorylation in COLXVI cell clones. Indeed, immunoblots showed markedly higher ILK phosphorylation in the COLXVI cell clones than in the mock control cells (figure 1D). To verify that ILK is responsible for MMP9 induction, the protein was inhibited with the potent ILK inhibitor Cpd 22. Quantitative RT-PCR revealed that inhibition of ILK led to a 60%–80% decrease in MMP9 expression (figure 1E). In addition, gelatine zymography of cell supernatants showed a strong decrease in MMP9 secretion into the cell culture supernatant (figure 1F). A luciferase activity assay, which was performed with a vector containing a 930-bp promoter fragment cloned adjacent to a luciferase reporter, also resulted in a ∼40% decrease in MMP9 promoter activity after inhibition of ILK (figure 1G).

### Collagen XVI overexpression leads to activation of PKB/Akt

It is known that PKB/Akt is activated downstream of ILK. Therefore, we analyzed whether collagen XVI overexpression activated PKB/Akt. Figure 1H shows a profound increase of PKB/Akt phosphorylation in COLXVI cell clones compared to mock control cells. Our observation of higher PKB/Akt phosphorylation in COLXVI cell clones compared to mock controls is compliant to activation of ILK.

### Collagen XVI overexpression leads to an increase of ILK/kindlin-1 interaction

**Figure 2:**
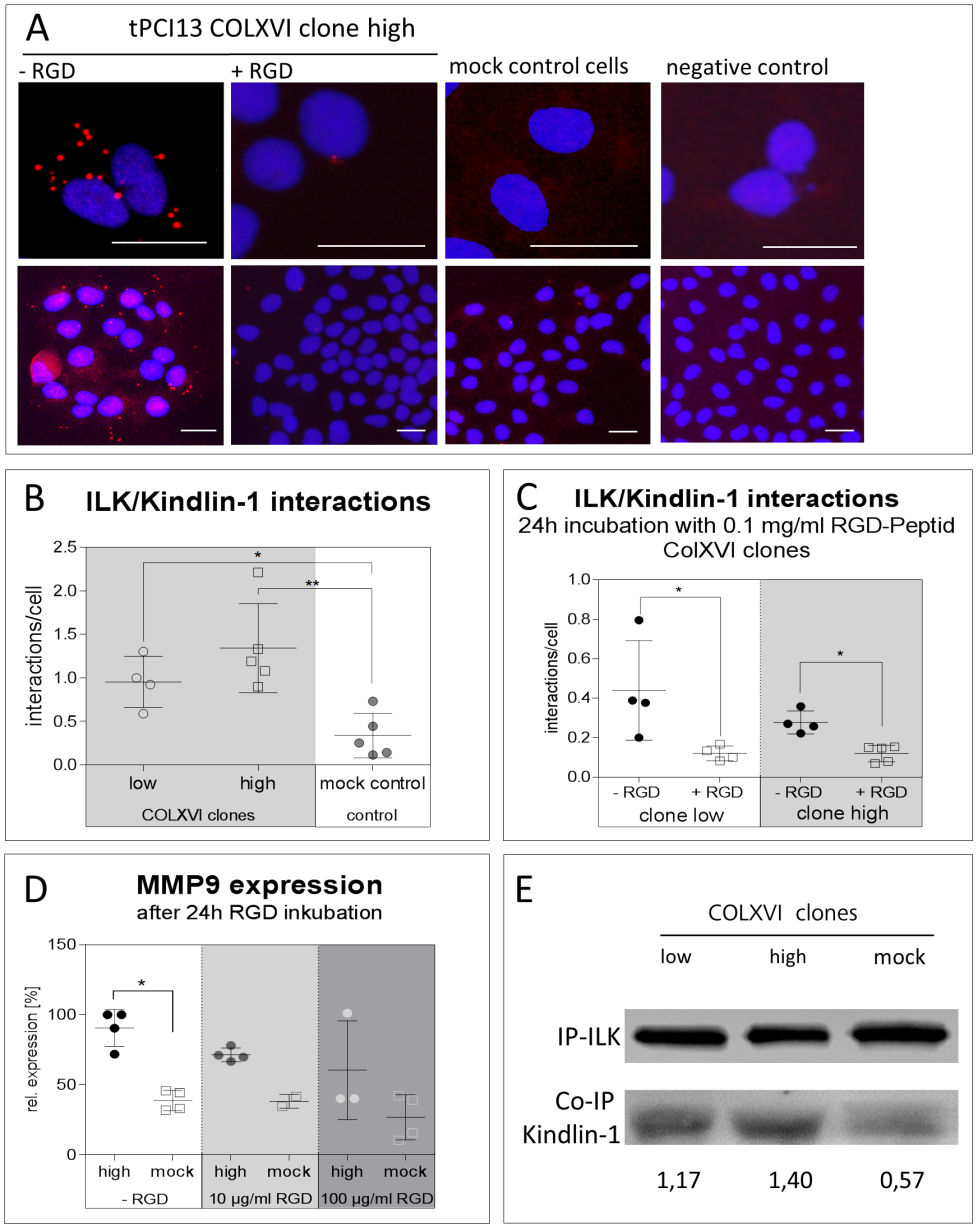
Collagen XVI overexpression leads to an increase of ILK/kindlin-1 interaction. (**A**) Proximity ligation assay for the analysis of ILK/kindlin-1 interaction in COLXVI and mock control cells (scale bar equals 50 µm). COLXVI cell clones exhibit an increased interaction of kindlin-1 and ILK at focal adhesions compared to mock control cells (**A+B**). After inhibition of focal adhesion formation with soluble RGD peptides (c  =  100 µg/mL) ILK/kindlin-1 interaction was significantly reduced (p<0.001, n = 100) (A+**C**). (**D**) Quantitative PCR of *MMP9* gene expression in COLXVI cell clones and mocks after 24 h incubation with soluble RGD peptides. Inhibition of focal adhesions via soluble RGD-peptides resulted in a dose-dependent decrease in *MMP9* gene expression (n = 3). (**E**) Co-Immunoprecipitation of ILK and kindlin-1 of protein extracts isolated from COLXVI clones and mocks. COLXVI cell clones exhibit an increased ILK/kindlin-1 interaction depending on collagen XVI dose. Mock control cells showed least ILK/kindlin-1 interaction.

Kindlin-1 is overexpressed in COLXVI cell clones [12]. Therefore, we tested if kindlin-1 interacts with ILK in our model. The proximity ligation assay shows a clear interaction of ILK with kindlin-1 in all observed COLXVI cell clones. Figure 2A (panel 1) depicts the interactions of the COLXVI cell clone with the highest collagen XVI expression. In mock control cells, there was only very weak or no ILK/kindlin-1 interaction. Figure 2B shows the quantification of ILK/kindlin-1 interaction as described in Materials and Methods. COLXVI cell clones show on average 2.5 interactions per cell, whereas mock controls interact on average 0.5 times per cell. Immunofluorescence images indicated that ILK/kindlin-1 interactions were only observed at focal adhesion contacts (figure 2A). This was confirmed by inhibition of the formation of FAs with soluble arginine-glycine-aspartate (RGD) peptides. Strikingly, quantification of the proximity ligation assay in figure 2C revealed a significant decrease in ILK/kindlin-1 interaction in COLXVI cell clones (p<0.01) treated with 100 µg/mL soluble RGD peptides. These data suggest that collagen XVI induces ILK/kindlin-1 interaction. Further, decrease in MMP9 gene expression was found to be dependent on the amount of soluble RGD-peptides employed to inhibit focal adhesions (figure 2D). The co-immunoprecipitation experiment with kindlin-1 and ILK in figure 2E corroborates our findings. There were similar levels of ILK expression in low and high expressing COLXVI clones and mock controls. However, immunodetection of kindlin-1 in these precipitates revealed a collagen XVI dose dependent ILK/kindlin-1 interaction with mock controls showing least interaction.

### The AP-1 binding site at –98bp is important for collagen XVI dependent MMP9 induction

**Figure 3:**
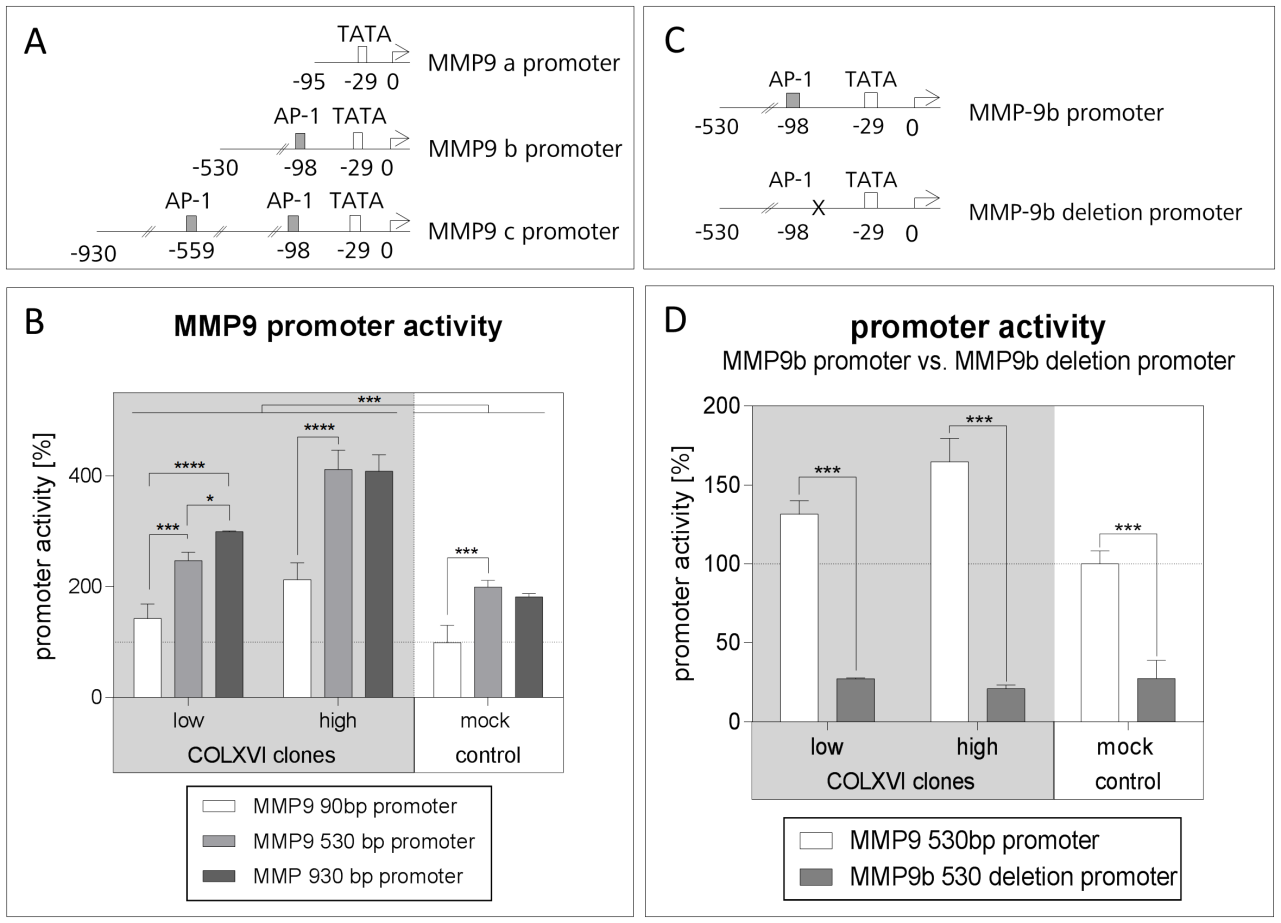
The AP-1 binding site at –98bp is important for collagen XVI dependent MMP9 induction. (**A**) Schematic presentation of the three different MMP9 promoter fragments. MMP9a, MMP9b, and MMP9c contain 0, 1, and 2 AP-1 binding sites, respectively. (**B**) Comparison of MMP9 promoter activities of MMP9a, MMP9b, and MMP9c in COLXVI and control cells, respectively. The shortest MMP9 promoter fragment (MMP9a) exhibits the lowest activation. In general, COLXVI clones reveal a higher activation of the *MMP9* promoter than the mock control cells. The luciferase reporter shows increased activation of the *MMP9* promoter in clone high compared to clone low (n = 3). (**C**) Schematic presentation of the MMP9b promoter and the MMP9b deletion promoter, respectively. The MMP9b deletion promoter does not contain the AP-1 binding site 98 bp upstream from the start codon. (**D**) Promoter activity of the MMP9b promoter after deletion of the AP-1 binding site. After deletion of the AP-1 binding site the MMP9b promoter activity decreases significantly (n = 3).

To identify the promoter region of MMP9 responsible for collagen XVI-dependent induction, we cloned MMP9 promoter fragments (90 bp, 530 bp and 930 bp upstream of the MMP9 start codon, respectively) adjacent to a luciferase reporter gene. The 930-bp promoter region contained two AP-1 binding sites, the 530-bp region a single AP-1 site, while the 90-bp region contained no AP-1 binding site (figure 3A). These constructs were each transfected into our COLXVI and mock cell clones. Compared to the COLXVI low expressing and the mock control cell clones, MMP9 promoter activity of the 930-bp and 530-bp regions was twice as high in the COLXVI high expressing cell clone. In addition, the two longer promoter fragments revealed higher luminescence intensity than the 90-bp fragment (figure 3B).

Since the two longer promoter fragments exhibited higher reporter activity than the 90-bp fragment containing no AP-1 binding site, we concluded that AP-1 was involved in MMP9 induction.

The additional AP-1 site in the 930-bp fragment did not cause a further increase in luciferase reporter signal over that of the 530-bp fragment containing only the AP-1 site at –98bp. Therefore, we hypothesized that the latter was solely responsible for MMP9 induction (figure 3B). To test this assumption we deleted the AP-1 binding site in the 530 bp fragment (figure 3C). This led to a 4-fold decrease in luciferase activity, thus confirming that the AP-1 site at -98 bp was indeed responsible for MMP9 induction (figure 3D).

### COLXVI cell clones show an interaction of c-Fos and dyskerin together with an increased nuclear JunB localization

**Figure 4:**
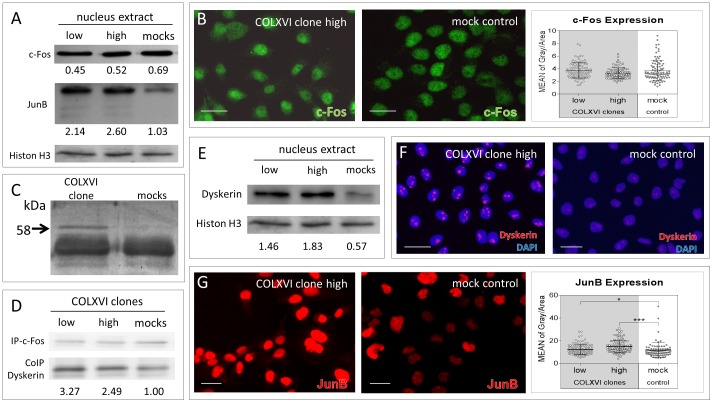
Collagen XVI overexpression leads to c-Fos – dyskerin interaction and increased nuclear JunB localization. (**A**) Immunoblot analyses of JunB and c-Fos nuclear extracts from COLXVI cell clones and mock control cells. COLXVI cell clones show a higher amount of JunB compared to mock control cells. COLXVI cell clones do not differ from mock control cells in c-Fos expression. (**B**) Immunofluorescence staining of c-Fos (green) in COLXVI cell clones and mock control cells (scale bar equals 50 µm). COLXVI cell clones do not differ from mock control cells in their c-Fos expression. Quantification was performed measuring the fluorescent intensity of 100 COLXVI cells and mock controls, each. (**C**) Silver stained gel of protein lysates from COLXVI cell clones and mock control cells, respectively, after immune precipitation of c-Fos. In COLXVI cell clones a band with a size of 58 kDa was differentially expressed. Mass spectrometry revealed it as dyskerin. (**D**) Co-immunoprecipitation of c-Fos and dyskerin of protein extracts isolated from COLXVI clones and mocks. COLXVI cell clones exhibit an increased interaction of c-Fos and dyskerin compared to mock control cells. (E) Dyskerin immunoblot of nuclear extracts from COLXVI cell clones and mock control cells. COLXVI cell clones exhibit a higher protein amount of dyskerin than mock control cells. (**F**) Immunofluorescence staining of dyskerin (red) in COLXVI cell clones and mock control cells (scale bar equals 50 µm). COLXVI cell clones showed strong dyskerin staining compared to mock control cells (**G**). Immunofluorescence staining of JunB (red) in COLXVI clones and mock control cells (scale bar equals 50 µm). COLXVI cell clones exhibit stronger JunB staining compared to mock control cells. Quantification of signal intensity demonstrates significantly increased nuclear JunB expression in COLXVI cells compared to mock controls.

We performed RT-PCR experiments to screen for the most abundantly expressed AP-1 protein isoforms in our cell lines. Among all tested AP-1 family members (FOS, JUNB, JUND; FRA1, FRA2), FOS and JUNB showed robust expression levels. Expression of c­Fos has been reported to be associated with MMP9 expression [19]. Since our data implied involvement of AP-1 in collagen XVI-induced MMP9 expression, we compared the c-Fos protein expression in COLXVI cell clones with mock control cells. Analysis of nuclear extracts did not show any differences in c-Fos expression between COLXVI cell clones and mock controls (figure 4A first row). Immuno­fluorescence analysis also failed to reveal any difference in the nuclear amount of c-Fos expression in COLXVI cell clones compared to mock controls (figure 4B, images). Semiquantitative evaluation confirmed these data (figure 4B). Next we searched for interaction partners of c-Fos in COLXVI cells vs mock controls. To that end, we performed an immunoprecipitation experiment with subsequent reducing SDS-PAGE electrophoresis of the immunoprecipitate. A protein band with a molecular weight of 58 kDa distinguished COLXVI cells from control cells (figure 4C). Tandem mass spectrometry of a tryptic digest of this band revealed the protein dyskerin as being the differential interaction partner of c-Fos in COLXVI cells. In comparison, control cells showed only a very faint interaction of c-Fos with dyskerin. Co-immunoprecipitation experiments confirmed these findings (figure 4D) indicating an increased c-Fos/dyskerin interaction in COLXVI clones in contrast to mock control cells. Immunoblot analyses and immunofluorescence staining corroborated these data, showing increased nuclear dyskerin expression in COLXVI cells compared to mock control cells (figure 4 E and F).

Western blot analysis of JunB protein expression revealed a clear upregulation in nuclear JunB expression in COLXVI cells (figure 4A, second row). We had not observed an upregulation of JUNB at the mRNA level (data not shown). Immunofluorescence staining of COLXVI cells and control cells confirmed the significant increase in nuclear JunB protein expression in COLXVI cells compared to mock controls (figure 4G). These data suggest a clear difference in AP-1 protein distribution and protein interaction in COLXVI cells compared to mock control cells.

### Inhibition of AP-1 leads to downregulation of MMP9 expression

**Figure 5:**
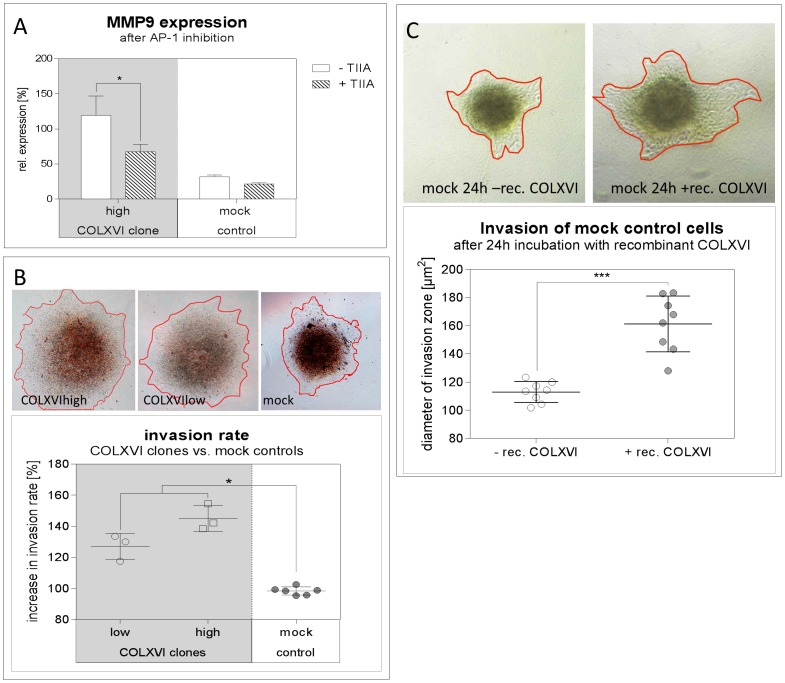
Collagen XVI overexpression leads to increased invasion of OSCC cells. (**A**) Quantitative PCR of *MMP9* gene expression of COLXVI and mock control cells after AP-1 inhibition with Tanshinone IIA (TIIA; c  =  100 ng/mL). The expression of *MMP9* decreased in the COLXVI cell clones after AP-1 inhibition with Tanshinone IIA. (n = 3) (**B**) 3D micromass pellets of COLXVI cells compared to mock control cells 24 h after placement. COLXVI cells (high and low expressing) show a significantly wider invasion zone (orange line) compared to mock control cells (p<0.01; n = 10). (**C**) 3D micromass pellets of mock control cells after 24 h of incubation with 500 ng/mL recombinant collagen XVI. Incubation with recombinant collagen XVI resulted in a significantly increased spreading of the invasion zone (orange line) compared to the control (p<0.001; n = 10).

To test whether AP-1 participates in the regulation of collagen XVI-dependent MMP9 gene expression we inhibited AP-1 with Tanshinone IIA. A significant decrease in MMP9 gene expression was observed in COLXVI cells. We did not observe a significant change in MMP9 gene expression in mock control cells after inhibition with Tanshinone IIA (figure 5 A).

### Collagen XVI overexpression leads to an increased invasion of OSCC cells

To analyze if collagen XVI-induced MMP9 expression leads to OSCC cell invasion, we generated 3D micromass pellets of COLXVI cells and mock controls and embedded them in partially degraded collagen I gel. After 48 h, COLXVI cells yielded a significantly larger invasion zone compared to the mock controls (figure 5B). The cores of the COLXVI cell 3D micromass pellets were more diffuse than those of the mock control cells and the invasion zones were significantly wider. Moreover, we observed a sheet like invasion pattern of COLXVI cells, while mock controls exhibited a rather diffuse cellular invasion pattern (figure 5B).

To ascertain that exogenous treatment of OSCC cells with excess of recombinant collagen XVI also resulted in an increased invasion we added recombinant collagen XVI at a concentration of 500 ng/mL during the generation of mock control micromass pellets. Again, the areas of the invaded spheroids in partially degraded collagen I matrix were determined after 48 h. Interestingly, in COLXVI cell clones we observed a profoundly higher invasion in the partially degraded collagen I matrix compared to mock control cells without exogenous collagen XVI (figure 5C)

## Discussion

In normal oral epithelium as in the dermis, collagen XVI is predominantly located at the dermal-epidermal junction [8], [16]. Collagen XVI is involved in dermal architecture by interacting with integrins alpha1beta1, alpha2beta1 and the microfibrillar apparatus [8], [20]. Collagen XVI also plays a role in tumorigenesis. It was demonstrated, that collagen XVI expression is up-regulated in glioblastoma [14] and its inhibition leads to reduced glioma invasiveness [16]. Dysplastic stages of OSCC exhibit an excessive amount of collagen XVI [12]. Our group has shown that collagen XVI overexpression in OSCC facilitates early S-phase entry [12]. The present data suggest in addition, that collagen XVI induces *MMP9* expression via the specific AP-1 promoter region located 98 bp upstream of the start codon of *MMP9*. We noticed a concurrence between the amount of collagen XVI protein expression and *MMP9* promoter induction, as the cell clones with most abundant collagen XVI expression tended to induce the *MMP9* promoter stronger than cell clones with less collagen XVI expression. We detected a strong gelatinolytic activity at 88 kDa and 92 kDa in oral squamous cell carcinoma cells treated with a high concentration of recombinant collagen XVI. These bands correspond to the activated and pro (latent) forms of MMP9, respectively. Increased expression of MMP9 has been associated with the acquisition of an invasive phenotype in many tumors. *MMP9* is activated early during melanoma progression and is secreted by keratinocytes [21]. In tongue squamous cell carcinoma high MMP9 expression is observed in neoplastic cells at the invasion front [22]. In OSCC, MMP9 serves as a predictor of tumor recurrence when detected in histologically-negative surgical margins of OSCC [23]. Moreover, a direct link was shown by a strong reduction of invasion and adhesion of ovarian cancer cells after treatment with MMP-9 siRNA [24]. Furthermore, oral squamous cell carcinoma cells exhibited reduced invasion after suppression of MMP9 via ESE-1 [25] and Heikkilä et al. illustrated that selective antigelatinolytic peptides specifically designed against MMP2 and MMP9 clearly inhibited invasion of tongue squamous cell carcinoma cells [26]. Moreover, we observed a differential upregulation of the AP-1 family members JunB and c-Fos in COLXVI cells compared to mock control cells. AP-1 family members have been implicated in oral carcinogenesis [27]. Mishra et al. could show that precancerous cases exhibit JunD homodimers, whereas c-Fos/JunD was the most prevalent complex found in cancer tissues. Further, c-Fos showed a specific interaction with dyskerin (DKC1), an important component of the telomerase complex in OSCC cells. This interaction has not yet been described in the literature but Sachdev et al. found a gradual increase of c-Fos expression in the oral cavity from normal oral mucosa to premalignant lesions to squamous cell carcinoma [28]. Moreover, comparing different stages of oral carcinogenesis in 100 oral tissue specimen Mishra et al. described a constitutive activation of AP-1 and a concomitant upregulated expression of the majority of AP-1 family of proteins and mRNA [27]. Overexpression of dyskerin has been detected in oral squamous cell carcinoma and in immortalized and transformed keratinocytes [29]. In accordance with Alawi et al., upregulation of dyskerin correlates with increased cell proliferation rate and tumor growth [29].

As for JunB, Wang et al. demonstrated that the expression level of JunB significantly increased in human colorectal adenocarcinomas [30]. Furthermore, the expression of JunB is enhanced in 4-nitroquinoline 1-oxide induced rat tongue cancers [31]. These data fit well with the upregulation of JunB in our COLXVI cells and underline the supporting role of collagen XVI during the progression of OSCC. Furthermore, we have observed a strong activation of ILK and PKB/Akt in COLXVI cells. Inhibition of ILK led to the downregulation of MMP9 protein expression and secretion, suggesting a direct link between ILK-signaling and *MMP9* expression. Inhibition of ILK also led to a strong reduction of MMP2 expression and activity, suggesting the existence of a general ILK-dependent mechanism underlying MMP regulation. Toussard et al. have shown, that ILK overexpression in intestinal and mammary epithelia cells results in an upregulation of MMP9 secretion and therefore in a highly invasive phenotype. Inhibition of ILK leads to a decreased activation of AP-1 and deceased MMP9 promoter activity [32]. This is consistent with our data. Increased ILK expression correlates strongly with OSCC tumor invasion, higher tumor grade, advanced clinical stage, positive lymph node status and increased risk of recurrence [33]. Moreover, PKB/Akt activation contributes to a worse outcome in patients with tongue cancer [34]. Our previous data showed an upregulation of kindlin-1 in COLXVI cells [12]. Kindlin-1 has been observed to regulate polarity, proliferation and motility in epidermal keratinocytes, and it is required for RhoGTPase-mediated lamellipodia formation in keratinocytes [35]. Here, we demonstrated that COLXVI cells exhibited a significant amount of ILK/kindlin-1 interaction compared to the control cells at focal adhesion (FA) contacts. Blocking FAs via soluble RGD peptides [36] led to a significant decrease of ILK/kindlin-1 interaction and caused a downregulation of *MMP9* gene expression, suggesting that this interaction plays a critical role in AP-1 induced *MMP9* induction. We have already found putative integrin binding domains for collagen XVI [20]. These domains do not contain RGD sequences. Therefore, it is unlikely that the observed effect of diminishing ILK/kindlin-1 interaction is due to a displacement of collagen XVI by soluble RGD peptides. Montanez et al. revealed an interaction between Kindlin-2 and ILK in mouse fibroblasts and proposed that Kindlin-2 is required for ILK localization in FAs [37]. In summary, our study demonstrates a strong impact of abundant collagen XVI expression on the induction of *MMP9* in OSCC cells. This induction occurs via increased phosphorylation of ILK and PKB/AKT together with enhanced ILK/kindlin-1 interaction. Additionally, collagen XVI leads to an alteration of interaction partners and expression of AP-1 proteins. This makes collagen XVI a possible molecular target for optimized future cancer therapies and even a suitable diagnostic tool.

## Materials and Methods

### Cell lines and culture conditions

The human OSCC cell line PCI13 was kindly provided by Prof. T.L.Whiteside (University of Pittsburgh Cancer Institute (PCI), Pittsburgh, PA). The cell line was established from primary tumors in the laboratory at the University of Pittsburgh as described previously [38]. The PCI13 cell line was established from a male patient, who suffered from low-grade OSCC of the retromolar triangle. The tumour stage was pT4pN1M0G3. The cells were maintained in DMEM containing 10% FCS (Invitrogen, Darmstadt, Germany), 2 mmol/L L-glutamine (PAA, Coelbe, Germany), and 1% penicillin/streptomycin (Sigma, Steinheim, Germany). Inhibition of ILK was performed with the ILK-Inhibitor Cpd 22 (Millipore, Billerica, USA, dissolved in DMSO (Sigma), stock concentration: 10 mM; concentration for inhibition: 300 nM). Inhibition of focal adhesion formation was performed with RGD-Peptides (Abbiotec, USA, dissolved in DMEM medium, stock concentration: 2 mg/ml). AP-1 was inhibited with Tanshinone II A (Torcis, UK, dissolved in DMSO (Sigma), stock concentration: 1 mg/ml; concentration for inhibition: 100 ng/ml)

### Cloning

For transfection experiments, PCI13 cells were transfected with the expression vector pCEP-Pu BM40SP C-StrepII [17], [38] using FuGene HD Transfection Reagent (Roche, Penzberg, Germany) to stably express recombinant collagen XVI. The cell clones were denominated COLXVI C1-4. The coding sequence was inserted between restriction sites NheI and NotI. Growth medium for transfected cells was DMEM, 10% FCS (Pan) supplemented with 0.25 µg/mL puromycin (Biomol, Hamburg, Germany). Stable mock control cell lines were transfected with pCEP-Pu BM40SP vector without the collagen XVI coding sequence. The mock controls were denominated mock1 and mock2. Recombinant collagen XVI was purified from culture medium by means of one-step gravity flow affinity chromatography using Strep-Tactin Superflow (IBA, Göttingen, Germany) columns as described in Kassner et al. [17].

### Immunoblot analysis

Lysates were prepared using RIPA buffer (Sigma) containing protease inhibitor (Roche). The protein concentration of the cell lysates was determined by bicinchoninic acid assay (BCA, Pierce, Rockford, IL), stored at –20°C. 30 µg of total protein were separated by SDS-PAGE using a 10% resolving gel and transferred onto PVDF membrane. Blots were blocked in 3% BSA (Sigma) in PBS (Sigma) containing 0.1% Tween 20 (Sigma). Primary antibodies (with the exception of collagen XVI all from Santa Cruz, Dallas, TX): collagen XVI (1∶5,000, Sigma), ILK (1∶200), P-ILK (1∶200), c-Fos (1∶200), P-c-Fos (1∶200), Akt/PKB (1∶200), and P-Akt/PKB (1∶200). All appropriate secondary antibodies conjugated with horseradish peroxidase were purchased from Pierce. For protein visualization Roti Lumin kit (Roth, Karlsruhe, Germany) was used. Equal loading was verified with mouse antibody against beta-actin (1∶10,000, Abcam, Cambridge, UK). Semiquantitative analysis in figure 4 was performed by referring the band intensity to Histone H3. All experiments were repeated at least 3 times.

### Co-immunoprecipitation

Lysates were prepared using RIPA buffer (Sigma) containing protease inhibitor (Roche). The protein concentration of the cell lysates was determined by bicinchoninic acid assay (BCA, Pierce, Rockford, IL), stored at –20°C. 200 µg of total protein were immune precipitated by µMACS™ Protein A/G MicroBeads MultiMACS™ Protein A/G Kit (Miltenyi Biotec, Bergisch Gladbach, Germany) and performed according to the manufacturer’s instructions. The following immunoblot analyses were performed as described above. Antibodies for immunoprecipitation: ILK (4 µg per immunoprecipitation; SantaCruz), c-Fos (2 µg per immunoprecipitation; SantaCruz). Primary antibodies: ILK (1:200; SantaCruz), c-Fos (1∶200; SantaCruz), kindlin-1 (1∶500; polyclonal rabbit; Abcam), dyskerin (1∶200; SantaCruz)

### Gelatine zymography

For gelatine zymography, cells were cultured for 24 h without FCS. Supernatants were harvested and concentrated with centrifugal filters (Amicon, Tullagreen, Ireland). Aliquots of supernatants, containing an amount of 2 µg of total protein, were mixed with equal volumes of two-fold concentrated sample loading buffer (2 mM EDTA, 2% SDS, 20% glycerol, 0.02% bromophenol blue, 20 mM Tris/HCl, pH 8.0) and subjected to electrophoresis in 10% SDS-polyacrylamide gels containing 1% gelatine. Subsequently, gels were washed twice for 30 min in 2.5% Triton-X 100, rinsed in distilled water, and developed for 48 h at 37°C in 50 mM Tris/HCl, pH 8.5, containing 5 mM CaCl_2_. Finally, the gels were stained with Coomassie Brilliant Blue R250 (Serva, Germany) to visualize protease activity and were photographed.

### Immunocytochemistry

Cells were plated on 8 ChamberSlides (BD, Franklin Lakes, NJ) and fixed with 4% paraformaldehyde for 10 min. Cultured cells were washed with PBS and blocked with 3% (v/v) normal goat serum (Sigma) in PBS for 60 min at 37°C. Appropriate first antibodies were diluted in 1.5% (v/v) normal goat serum (Sigma) and incubated over night at 4°C: c-Fos (polyclonal; rabbit IgG: 2 µg/mL, Santa Cruz sc-52); JunB (monoclonal; mouse IgG1: 2 mg/mL, Santa Cruz sc-8051); dyskerin (monoclonal; mouse IgG1: 2 µg/mL, Santa Cruz sc-365731). The secondary antibody was diluted in 1.5% (v/v) normal goat serum (Sigma) in PBS and incubated at 37°C for 1 h. For immunocytochemistry, secondary antibodies linked to fluorescent dyes AlexaFluor 488 and AlexaFluor 568 (Molecular Probes, Eugene, OR), respectively, were used: Goat anti-rabbit AlexaFluor 488: 1∶500; goat anti-mouse AlexaFluor 568: 1∶500. Nuclei were counter-stained with 300 nM DAPI (Molecular Probes) for 10 min. To quantify c-Fos and JunB expression, the mean of grey for each cell (n = 100) was measured using the open-source software ImageJ 1.46r [39] and divided by the cell area.

### Proximity ligation assay

Interaction of kindlin-1 and integrin-linked kinase (ILK) was analysed by means of a proximity ligation assay (Duolink®, Eurogentec, Seraing, Belgium) performed according to the manufacturer’s instructions [40]. First antibodies: ILK (polyclonal goat; Santa Cruz), kindlin-1 (polyclonal rabbit; Abcam). An antibody against the N-terminus of integrin beta (Millipore, Billerica, MA) served as negative control. Secondary antibodies were used against rabbit (plus-DNA-strand) and goat (minus-DNA-strand), respectively. First antibodies: Appropriate first antibodies were diluted in blocking buffer 1h at 37°C in a humid chamber. PLA Probes: Anti-goat minus dilution 1∶5. Anti-rabbit plus dilution 1∶5 incubated 1 h at 37°C in a humid chamber. Ligation: 30 min at 37°C in a humid chamber. Amplification: 120 min at 37°C in a humid chamber. For the quantification of the kindlin-1 – ILK interaction, interactions were counted and divided by the cell number (n = 100) in each picture. Cells were counted in five pictures with a magnification of 400x. Each data point represents the average interactions per cells for one image.

### RNA isolation and quantitative real-time polymerase chain reaction

Total cellular RNA was isolated using the RNeasy mini kit (Qiagen, Hilden, Germany) according to the manufacturer’s instructions. Reverse transcription of 1 µg RNA to complementary DNA (cDNA) was performed using transcriptor high fidelity cDNA synthesis kit (Roche) according to the manufacturer’s protocol. cDNA was amplified using the Brilliant III ultra fast quantitative polymerase chain reaction master mix (Stratagene Agilent Technologies, Santa Clara, CA) in combination with TaqMan UPL probes (Roche). Real-time PCR primers were obtained from Eurofins (Ebersberg, Germany). 18S mRNA was used for normalization. Primers: 18s: 5′- GCAATTATTCCCCATGAACG-3′ and 5′-GGGACTTAATCAACGCAAGC-3′, probe #48. *MMP9*: 5′-TTCGACGATGACGAGTTGTG-3′ and 5′-TCCAAACCGAGTTGGAACC-3′, probe: #61. *FOS*: 5′-GGGGCAAGGTGGAACAGT-3′ and 5′-TCTCCGCTTGGAGTGTATCA-3′, probe: #24. *JUNB*: 5′- CAAGGTGAAGACGCTCAAGG-3′ and 5′-TCATGACCTTCTGTTTGAGCTG-3′, probe #32.

### Luciferase activity assay

Linked to the luciferase gene, the different promoter fragments of *MMP9* were cloned in the pGL4.16 (luc2/Neo) vector (Promega, Fitchburg, WI) according to the manufacturer’s instructions. The control vector was pGL4.74 (hRluc/TK) (Promega). The COLXVI cells were transiently transfected with FuGene HD Transfection Reagent (Roche) with the described vectors. The luciferase activity was measured using the Dual-Luciferase® Reporter Assay System (Promega) according to the manufactureŕs instructions. To design the cloning promoters the sequence of the *MMP9* transcript ENST00000372330 from Ensembl.org (accessed 2013 Dec 20) was used. Promoter primers: 90-bp promoter: 5′-TACATTTACATTGGTACCAGCACTTGCCTGTCAAGGA-3′ and 5′- TTGATACTCGAGCCAGCACCAGGAGCACC-3′; 530-bp promoter: 5′-TACATTGGTACCAAAGAGGACAGAGCCTGGA-3′ and 5′- TTGATACTCGAGCCAGCACCAGGAGCACC-3′; 930-bp promoter: 5-‘tacattGGTACCTCTTGGGTCTTGGCCTTAGT-3′ and 5′- TTGATACTCGAGCCAGCACCAGGAGCACC-3′.

### Invasion Assay

For invasion measurements 3D micromass pellets were generated. For each 3D micromass pellet, 400 cells were seeded for 24 h in cone-shaped 96-well plates (Nunc, Germany). The 3D micromass pellets were then placed in degenerated Collagen I matrix (PureCol, Advanced BioMatrix, San Diego, CA; 1 mg/mL, pH 7.5) and cultured for 48 h. The 3D micropelletes were photographed before and 48h after cultivation in degenerated Collagen I matrix. The area of the invasion zone was measured using Photoshop CS4.

### Liquid chromatography-tandem mass spectrometry

COLXVI cells and mock control cells were synchronized and subsequently cultured in DMEM medium (10% FCS, 1% Penicillin/Streptavidin, 2 mmol/L L-Glutamin) for 24 h. Afterwards, cell lysates were prepared using RIPA buffer. 200 µg of total protein were immunoprecipitated according to the manufacturer’s instructions (Miltenyi Biotec; µMACS™ Protein A/G MicroBeads) with the appropriate antibody. The precipitates were separated by SDS-PAGE. Gels were stained with EZ blue gel staining reagent (Sigma-Aldrich) for 16 h at room temperature. Afterwards, respective bands were excised and gel pieces were washed three times alternately with 50 µL 50 mM NH_4_HCO_3_ and 25 mM NH_4_HCO_3_ in 50% acetonitrile (Merck, Darmstadt, Germany). Subsequently, the gel slices were dried in a vacuum centrifuge. 5 µL of trypsin solution (Promega, Mannheim, Germany) (12.5 ng/µL in 50 mM ammoniumbicarbonate (AppliChem, Darmstadt, Germany)) were added to each gel piece and incubated at 37°C overnight for in-gel-digestion. The obtained peptides were eluted with 20 µL of 5% formic acid (Merck) and subjected to nano-LC-MS/MS-analysis. Nano-LC was performed on an Ultimate 3000 nano-HPLC-system (Dionex GmbH, Idstein, Germany). Samples were pre-concentrated on a 2 cm x 100 µm I.D. C18-column (nanoseparations, Nieuwkoop, The Netherlands) using 0.1% trifluoroacetic acid (Merck) at a flow-rate of 8 µL/min. The peptides were then separated on a 15 cm×75 µm I.D. C18-PepMap-column (flow-rate 300 µL/min; Dionex GmbH, Idstein, Germany) using a 1 h binary gradient from 5-50% solvent B (solvent A: 0.1% formic acid; solvent B: 0.1% formic acid/84% acetonitrile). The nano-HPLC was coupled directly to a QTOF mass spectrometer (QStar XL, AB Sciex, Darmstadt, Germany) acquiring repeatedly one full-MS and two tandem-MS-spectra of the most intensive ions in the respective full MS-scan. The tandem-MS-spectra were searched against the Uniprot-database using the Mascot Daemon and the Mascot algorithm (version 2.1; Matrix Science Ltd., London, UK) with the following adjustments: taxonomy: *Homo sapiens*, trypsin as protease, max. one missed cleavage site, oxidation of methionine, pGlu for N-terminal Gln as variable modifications, 0.2 Da-tolerance for MS and MS/MS-signals, only doubly and triply charged ions. Only proteins, for which at least two different peptides could be identified in significantly scored spectra after manual verification, were considered.

### Statistical analysis

Significances were determined with assistance of GraphPadPrism V.6 using Mann Whitney U test. P<0.05 was considered as significant. Error bars represent standard deviation.
